# Environment sensing and response mediated by ABC transporters

**DOI:** 10.1186/1471-2164-12-S1-S8

**Published:** 2011-06-15

**Authors:** Sarah E Giuliani, Ashley M Frank, Danielle M Corgliano, Catherine Seifert, Loren Hauser, Frank R Collart

**Affiliations:** 1 Biosciences Division, Argonne National Laboratory, Lemont, IL 60490, USA; 2 Biosciences Division, Oak Ridge National Laboratory, Oak Ridge, TN 37831, USA

## Abstract

**Background:**

Transporter proteins are one of an organism’s primary interfaces with the environment. The expressed set of transporters mediates cellular metabolic capabilities and influences signal transduction pathways and regulatory networks. The functional annotation of most transporters is currently limited to general classification into families. The development of capabilities to map ligands with specific transporters would improve our knowledge of the function of these proteins, improve the annotation of related genomes, and facilitate predictions for their role in cellular responses to environmental changes.

**Results:**

To improve the utility of the functional annotation for ABC transporters, we expressed and purified the set of solute binding proteins from *Rhodopseudomonas palustris* and characterized their ligand-binding specificity. Our approach utilized ligand libraries consisting of environmental and cellular metabolic compounds, and fluorescence thermal shift based high throughput ligand binding screens. This process resulted in the identification of specific binding ligands for approximately 64% of the purified and screened proteins. The collection of binding ligands is representative of common functionalities associated with many bacterial organisms as well as specific capabilities linked to the ecological niche occupied by *R. palustris*.

**Conclusion:**

The functional screen identified specific ligands that bound to ABC transporter periplasmic binding subunits from *R. palustris*. These assignments provide unique insight for the metabolic capabilities of this organism and are consistent with the ecological niche of strain isolation. This functional insight can be used to improve the annotation of related organisms and provides a route to evaluate the evolution of this important and diverse group of transporter proteins.

## Background

Bacterial transport proteins have an essential role in mediating the uptake and efflux of small molecules with the environment and the efflux of large molecules to the outer surface of the cell [1]. These proteins comprise a heterogeneous group representative of their diverse functional and cellular roles. Transporter proteins are clustered into approximately 600 different families based on the transporter classification system [2] that incorporates both phylogenetic and functional information. This system effectively organizes transporters to the superfamily or family level, but provides very limited insight into the specific ligands which are transported by these proteins. This lack of specific functional information limits our ability to link cellular metabolic capabilities with environmental signaling molecules or nutrients and generate predictive models for cellular response to environmental changes. Certainly, improved methods for functional characterization of ligands associated with the genomic set of transporters (transportome, [3]) would provide critical insight into cellular capabilities for utilizing environmental nutrients and extruding toxic compounds.

To evaluate the impact of improved functional annotation of transporter proteins, we applied a high throughput screening method for identification of protein-ligand interactions to map ligands with transporter proteins. As many transport-associated proteins are integral membrane proteins, we used solute binding subunits of the ABC transporter family as surrogates for determining specificity of these transporters. This approach was validated for a set of bacterial ABC transporters [4] and provided valuable insight into this biologically relevant class of proteins. The family of ABC transport systems is widely distributed in all three kingdoms of life and can transport a variety of substrates such as metals, small ions, mono- and oligosaccharides, peptides, amino acids, iron-siderophores, polyamines and vitamins. In bacteria, ABC uptake transporters typically consist of a combination of a solute binding, two integral membrane, and two ATPase subunits. ABC efflux pumps are thought to lack the traditional functionality associated with the solute binding subunit, but the associated proteins sometimes form larger complexes with membrane fusion proteins [ i.e. the HlyD subfamily proteins, transporter classification 8.A.1.3.1, [2] and Outer Membrane Factors (transporter classification 1.B.17 [2]].

In bacteria, the number of ABC type transport systems is generally related to genome size with approximately 2-5% of the genome encoding components of the ABC family transporters [5]. However some soil bacteria contain a relatively high number of these transporters representing 40-70% of the all transporter proteins encoded in the genome. These are essential for the utilization of environmental nutrients and may reflect the competitive aspect of nutrient acquisition in the soil environment. To evaluate functional diversity of these systems, we examined the transport capabilities for *Rhodopseudomonas palustris*, a metabolically versatile bacterium commonly found in soils and water. This organism has the ability to produce hydrogen, fix carbon dioxide, and biodegrade organic pollutants and plant-derived material. The *R. palustris* CGA009 strain contains ~ 500 transport proteins representing most major transporter classes. Included in this set are ABC transporter clusters and individual components identified by the TransportDB [6] that comprise approximately 87 distinct functional units. The ABC transporter gene set is comprised of ~350 genes representing a significant fraction (~41%) of the genome apportioned to transport capability. This organism has a diverse metabolic repertoire and characterization of the ABC transporter capabilities would provide useful insight into the metabolic and cellular capacity to utilize environmental nutrients and to extrude toxic compounds of this and related organisms.

## Results

### Target protein selection

The genome set of 105 candidate solute binding proteins (SBP’s) of ABC transporters in *R. palustris* were selected for production and functional characterization by ligand screening (see Additional file 1 for a list of ABC transporter proteins considered for this study). Most of the targets were extracted from TransportDB but the set was supplemented by our internal bioinformatic analysis of attributes such as genome context and protein sequence features. The prototypical periplasmic binding protein with a predicted periplasmic signal sequence comprised the majority of the target set. Seven targets did not have readily identifiable signal peptides or an N-terminal helical region (possible membrane anchor) but were included in the set based on either genome context suggesting the protein was part of an ABC transporter gene cluster (RPA1395, 2112, 2359, 2308, 4164) or from sequence homology predicting a periplasmic binding protein (PBP) domain (RPA4686, 1389, 3707). RPA3707 is annotated as a "nitrate transporter component nrtA" and categorized as an ATPase by TransportDB. Though it is part of an operon with the gene RPA3706, coding for a putative two-component response regulator antitermination factor NasT, it has a conserved PBP domain and thus was included in this target set. The experimental target set included several proteins having signal peptides or N-terminal helices which were predicted to be associated with efflux pumps. Three genes annotated as membrane fusion proteins (RPA0681, 1648 and 4088) are specific for efflux pumps and generally believed not to influence substrate specificity. These were included in the ligand screen on the basis of a recent study demonstrating metal-induced conformational changes in the ZneB protein which were suggested to indicate an active role of membrane fusion proteins in efflux resistance systems [7]. The addition of these targets to the study efflux pump associated proteins resulted in a total of 108 candidate binding proteins (BP’s) targeted for the protein production and ligand screening protocols.

Interestingly, of the 108 candidate BP’s, 21 were not clustered with an integral membrane and ATPase subunits (Additional file 1 and TransportDB) based on either proximity in genome and/or functional annotation (predicted substrate) from sequence homology. There were 72 total gene clusters having at least one representative of each ABC transporter component; of these, 9 transporters were associated with 2 SBP’s, 1 was associated with 3 SBP’s, 61 had one associated SBP. Four additional gene clusters were each indicated by associating one SBP with either an integral membrane or an ATPase subunit. One transporter (RPA2039) was predicted to have a fused integral membrane and solute binding subunits in a single polypeptide but was not included in the final list.

### Protein production and characterization

**Figure 1:**
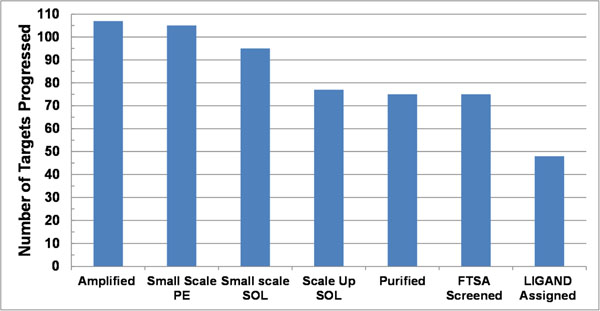
Experimental outcome from cloning to functional screening for the set of ABC transporter binding proteins from *R. palustris*.

One technical goal of this study was to benchmark the ability to clone, express and purify the genomic set of ABC transporter-associated solute binding proteins from *R. palustris* by applying a dual-vector expression strategy in the context of a high-throughput protein production process. The success rate for protein production steps (Fig. 1) is higher than reported for other typical genome scale productions experiments with bacterial proteins [8,9]. The working target set of 107 genes representing 99% of the total target set were those successfully amplified via PCR and annealed into a vector for heterologous expression in *E. coli*. The relatively high levels for production of soluble proteins are consistent with other studies on this family of proteins [10-12]. Overall, greatest attrition for cloned targets was associated with obtaining soluble protein; of the total target set, 97% expressed, but only 87% and 71% were soluble at small scale and large scale, respectively. All but two scaled soluble proteins were successfully purified and screened in the FTS assay. The final size of the screened set (69% of all targets or 75 proteins) is sufficient to provide a comprehensive assessment of the utility of the screening approach and provide insight into the nature of transport capabilities for this organism. Finally, ligand assignment was made to approximately 45% of the total protein set, which corresponds to 48 proteins or about 64% of the proteins assayed.

### Development of ligand library

The diversity of environmental and metabolic small molecule compounds is exemplified by the 16,000 biological compounds reported in KEGG database of known mapped metabolic pathways and cellular processes [13]. It is not currently possible to screen the complete set of candidate small molecule libraries but several strategies were invoked to enhance the ability to identify cognate ligands for the set of *R. palustris* binding proteins. One approach targeted common classes for all organisms (metals, vitamins, amino acids, peptides, and polyamines) and incorporated ligands known to bind this type of proteins. Another strategy examined the genome context of the transporter genes as these genes often cluster with known enzymes and could give an indication of metabolic capacity linked to transport. For example, select aromatic acid compounds such as benzoic acid, 4-hydroxybenzoic acid and p-coumaric acid were added to the SBP library, given knowledge of the ability of *R. palustris* to degrade aromatic acid compounds and the presence of a suite of genes involved in the degradation of benzoate and hydroxybenzoate (*3*, *4*). Results from initial binding outcomes of preliminary screens revealed the need to expand this class of ligands to probe binding specificity for similar compounds. A scan of the literature identified other similar aromatic acid compounds derived from plant lignin synthesis or breakdown such as m-coumaric acid, ferulic acid, cinnamic acid, syringic acid, quinic acid, benzylformic acid, and acetosyringone. Similarly, in response to assay detection of proteins’ affinity to benzoic acid, a set of related aromatic compounds was added, including salicylic acid, benzamide, benzylaldehyde, methylvanillate, phenylacetate, and others (*5*, *6*).

Clearly, various practical limitations prevent screening of all desired ligands, such as reagent unavailability, insolubility, toxicity, expensiveness, or incompatibility with the fluorescent dye in the assay. Despite this, an attempt was made to include at least one representative ligand for various categories of chemicals for which there is a known physiological or metabolic function in bacteria or which occurs naturally in the soil or freshwater environment of *R. palustris*. Ligand categories in the library were delineated by grouping compounds with similar chemical structure, resulting in about 33 distinct categories (Additional file 2).

### Mapping ligand and binding proteins in R. palustris

The fluorescence-based thermal shift assay was used in a microwell plate format to identify candidate binding ligands for a majority of the 108 ABC transporter candidate binding proteins (Table 1). Although a binding functionality is often inferred by the NCBI annotation, the screen was conducted in an unbiased manner in that each protein was screened against the bulk of the ligand library irrespective of the inferred function. In our analysis, a chemical was considered a “binding ligand” if the calculated ΔT_m_ (relative to the T_m_ without ligand) was 2° C or greater. The 2° C ΔT_m_ significance threshold is based on previous studies [4] for this family of proteins as well as a qualitative assessment of the observed variation for proteins between the various assays (individual ligand screens, pooled screens, and replicates). Multiple binding ligands were observed for some proteins and in most cases these ligands exhibited similar chemical properties such that a general ligand binding category could be assigned. For summation purposes, a maximum of four ligands (selected on the basis of highest ΔT_m_) is listed for each target in Table 1 with the complete binding data available as additional material (for a complete list of binding ligands see Additional file 3).

Ligand binding profiles for various proteins screened in the FTS assay were linked to additional evidence which corroborated the functional assignments, including genome context, literature descriptions for potential transport roles in metabolic pathways, and sequence homology with known PDB structures of ligand-bound proteins. A “High” data verification assignment indicates the FTSA ligand binding result is supported by good quality data and by two or more external sources of functional validation (literature, genome context, annotation/description, PDB homology, etc.). A “Moderate” data verification assignment indicates the FTSA ligand binding result is supported by one external source of functional validation while a “Low” data verification assignment indicates simply high confidence FTSA ligand-mapping data.

The outcome of this screening approach is the identification of specific roles for transporter SBP’s. The spectrum of ligands mapped to the transporter binding proteins include common functionalities associated with many bacterial organisms as well as specific capabilities linked to the ecological niche that provide insight into the extraordinary metabolic diversity of *R. palustris*. The experimental results are reviewed as a summary of ligand-binding events observed for general ligand categories followed by an evaluation of the biological relevance for specific assignments.

#### Metal binding proteins

Metal binding was observed for eight proteins in the FTSA screen. This group represents a heterogeneous mix annotated as solute binding (RPA0860, RPA0884, RPA1385, RPA2410, and RPA4686), efflux pump associated (RPA0681 and RPA4088), or hypothetical (RPA4236) proteins. One protein in this cluster (RPA0860) was predicted as a metal binding protein by TransportDB (Table 1). For the most part, the predicted annotation was either nonspecific or inconsistent with the outcome of the FTSA screens. Two proteins (RPA0860 and RPA4686) were stabilized by copper, nickel, or zinc although the degree of stabilization was different. The RPA0860 protein shared domain conservation with the TroA superfamily of periplasmic metal binding proteins that share a distinct fold and metal binding properties. This protein is homologous (e-value = 1 × 10^-35^) to a predicted periplasmic Zn^2+^ binding protein PsaA from *Streptococcus pneumoniae*. The crystal structure of this protein has been determined with bound zinc [14,15] and alignment of the RPA0860 protein sequence to this protein demonstrates good conservation of the residues for metal binding. Gene RPA4686 is not part of an ABC transporter operon, but its protein exhibits a similar metal binding profile as observed for the RPA0860 protein. The RPA4686 protein shares sequence similarity to several categories of periplasmic binding proteins but does not contain a recognizable periplasmic signal sequence. Although we observed specific stabilization of the protein with metal ions, there is little corroborative evidence to support the metal binding function or the association of this protein with a specific ABC transporter cluster.

The RPA0884 protein exhibited specific stabilization in the presence of Fe(III)/citrate and is a part of an ABC transporter cluster (Additional file 1). The genomic region adjacent to this transporter cluster contains several genes involved in the utilization of iron. The RPA0875 gene is annotated as a ferrocheletase, an enzyme that is present in most prokaryotes which catalyzes the insertion of ferrous iron into protoporphyrin IX to form protoheme IX (heme) [16]. The gene encoding the RPA0891 protein is annotated as the ferredoxin-containing subunit of a glutamate synthase complex.

The RPA1385 protein, which was incorrectly annotated as being a putative phosphonate transport system SBP, was shown to have a high affinity for binding vanadate. *R. palustris* is a nitrogen fixing bacteria and has been shown to utilize a vanadium nitrogenase (V-nitrogenase) as a metabolic alternative when molybdenum is limited in the environment [17]. The vanadium nitrogenase of *Azotobacter vinelandii* has been reported to catalyze both CO and N_2_ reductions and thus may provide a potential link between the evolution of carbon and nitrogen cycles [18]. Prior to this research, the cyanobacterium *Anabaena variabilis* (which also contains a V-nitrogenase) was the only organism know to contain a defined high-affinity vanadate transport system [18]. In *R. palustris*, genes RPA1381-1386 are annotated as components of a vanadate nitrogen fixation system based on homology to other similar proteins. However, in *R. palustris*, initial homology search approaches were unsuccessful in attempts to identify the high-affinity vanadate transport system. Subsequent annotation efforts have proposed vanadate transport for this system as inferred from substantial homology to ABC transporter genes in the same cluster in *A. vinelandii*. However, this update has not been successfully reflected in the current NCBI or JGI annotation lists. Our ligand mapping approach experimentally identifies the RPA1385 protein as the vanadate SBP gene for this ABC transport system. This finding not only identifies a key component of the vanadate nitrogenase fixation pathway for this organism, but may also confirm a proposed hypothesis for the presence of this system in *R. palustris* which suggests that vanadate transport systems have evolved at least twice from dissimilar ancestral genes [19].

Several other screened proteins were identified as metal binding proteins but little independent experimental evidence is available to support the functional assignments. The proteins encoded by the RPA2410 and RPA4236 genes exhibited stabilization by Cu^+2^ and Zn^+2^, respectively. Both proteins are part of a transporter cluster but there is little independent experimental evidence to support the functional assignments. The RPA0681 and RPA4088 genes are members of the HlyD family (membrane fusion protein, MFP superfamily) and are annotated as efflux pump components that link the ABC transporter in the plasma membrane with a pore in the outer membrane. Other MFP subfamilies specifically interact with other efflux pumps families such as the major facilitator superfamily (MFS) and resistance/nodulation/cell division (RND) family.

#### Binding proteins for aromatic compounds

**Figure 2:**
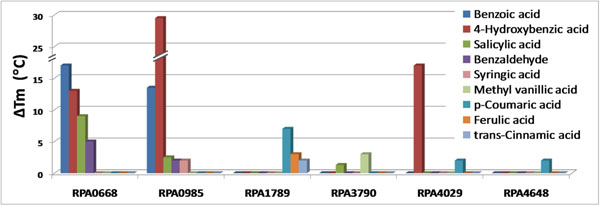
**Identification of solute-binding proteins in *R. palustris* which bind aromatic compounds. **A graph of the T_m_ shifts between protein with and without ligand displays six Solute-Binding Proteins which showed significant and specific stabilization with various structurally related aromatic compounds known as breakdown products of lignin in the soil environment.

Six proteins demonstrated binding to aromatic compounds as their primary ligand interaction. This activity was observed for five SBP’s (RPA0668, RPA0985, RPA1789, RPA4029, and RPA4648) and an efflux pump associated protein (RPA3790). Binding profiles of the SBP’s group further segregated this activity on the basis of proteins that bound benzene compounds with a single carboxyl group (RPA0668, RPA0985, and RPA4029) verses two proteins (RPA1789 and RPA4648) that bound benzene compounds with a propenoid side chain (3 carbons) rather than a single carboxyl group. The ligand profiles indicated that specificity was based on similar chemical structures for lignin degradation products such as benzoic acid and p-coumaric acid (Figure 2). In particular, RPA0668 (*hbaE*) displayed high affinity binding (ΔT_m_ = 17 °C) to benzoic acid and closely related derivatives, 4-hydroxybenzoic acid, salicylate, and benzaldehyde. The gene for this SBP is clustered with ABC transporter genes (*hbaEFGHI*) and localized between two well-known operons (*bad* and *hba*) for enzymes which are involved in the initiation of benzoic acid and 4-hydroxybenzoic acid anaerobic degradation via CoA ligation [20]. The FTS assay data is the first experimental validation demonstrating the involvement of this ABC transporter, via its associated SBP specificity, in the uptake and metabolism of benzoic acid and other aromatics.

Several other proteins exhibited specific binding of aromatic ligands and in several cases the ligand binding profiles were consistent with metabolic capabilities inferred from the *R. palustris* genome sequence. This organism contains several gene clusters implicated in the biodegradation of aromatic compounds [21]. Most notable are genes annotated to be involved in protocatechuate degradation (RPA4695-RPA4703, phenylacetate degradation (RPA1723, RPA1724, and RPA3765-3768), homoprotocatechuate degradation (RPA3755-RPA3762), and homogentisate degradation (RPA4670-RPA4675). The catalytic specificity of these enzymes has not been experimentally verified, but the metabolic capability generally overlaps with the observed transport profile.

Most notably, two SBP’s, RPA0985 and RPA4029, exhibited very high stabilization with 4-hydroxybenzoic acid having ΔT_m_’s of 29.5 and 17 °C respectively. Comparison of these two sequences using ClustalW revealed an overall significant identity (43%) and similarity (21%) of globally aligned residues. This is contrasted with alignments of RPA0985 and each of the other proteins in this group, where percent identity was less than 25%. In addition, alignment percent identity values displayed a significant positive correlation (R^2^ = 0.69) to the average T_m_ shift for the primary shared ligand between RPA0985 and each of the other five proteins. This suggests that there are homologous residues specific to ligand binding which discriminate even between ligands with similar structures (eg. benzoate and 4-hydroxybenzoate). Further structural studies are needed to differentiate between those residues specific for ligand binding and the general sequence signatures shared by periplasmic solute binding proteins. Overall, the FTS assay seems to be a good screening tool for determining relative affinities of a protein to similar ligands as well as comparing similar proteins with the same ligand, as demonstrated with this aromatic ligand-binding set of proteins.

Additionally, one protein bound p-coumaric acid, ferulic acid, and cinnamic acid with good affinities (Figure 2). The gene encoding this SBP (RPA1789) is located on the opposite strand but near an ABC transporter operon containing 3 genes: one containing an integral membrane subunit (RPA1793), and one containing an ATPase subunit (RPA1791) , and one containing fused integral membrane and ATPase subunits (RPA1792). Two genes which are in close proximity and on the same strand as the SBP encode the enzymes p-coumaric acid-CoA ligase (RPA1787) and p-coumaroyl hydratase/lyase (RPA1786). These enzymes have been predicted to catalyze the first two catabolic steps of p-coumaric acid degradation [22]. Previously, microarray transcriptome profiling and quantitative proteomics measurements were performed with *R. palustris* cells grown on p-coumaric acid, benzoic acid or succinic acid as the sole carbon source. These studies reported significantly increased protein abundances for the two enzymes and correlating increases with the ABC transporter SBP and ATPases for p-coumaric acid verses benzoic acid or succinic acid conditions. Based on this and the FTS assay data, we propose the function for this transporter is uptake of p-coumaric acid and related compounds in the environment. Additional evidence to support the function of this cluster includes a MarR transcriptional regulator (RPA1794) which shares a putative bidirectional promoter with the three gene ABC operon and may regulate the cluster; a phenylacetic acid degradation related protein (RPA1780); a 4-hydroxybenzoate hydroxylase (RPA1781) and a TrapT family transporter operon (RPA1782-4) that are known to transport carboxylates.

Results of FTSA-determined aromatic acid binding profiles for these 6 proteins are consistent with the knowledge that *R. palustris* grows well anaerobically and aerobically on cinnamic acid, ferulic acid, and 4-hydroxybenzoic acid, and anaerobically only on benzaldehyde, benzoic acid, and DL-mandelic acid, among other substrates tested [23]. Various substrates included in the FTSA ligand library but reported not to support *R. palustris* growth were ethyl-vanillic acid (methyl form in library), phenol, quinic acid, salicylic acid, shikimic acid and syringic acid. In light of this, it is interesting to note that assumed transport of a compound based on SBP/ligand interaction such as that of salicylic acid with proteins RPA0668, RPA0985 and RPA3790 may not necessarily be correlated with an organism’s ability to grow on that chemical. Still, this proposed suite of six ABC transporters for uptake of aromatic compounds in *R. palustris* expands the known list of bacterial ABC transporters which interact with aromatic compounds. The only family listed in the Transporter Classification Database http://www.tcdb.org that uptakes aromatic-type compounds is the Taurine Uptake Transporter (TauT) Family (3.A.1.17), including the aromatic sulfonate porter, SsuABC, of *Pseudomonas putida* and the phthalate uptake system, OphFGH, of *Burkholderia capacia*[24]. Other literature suggests homologous aromatic compound transport roles of branched-chain amino acid ABC transporters in aromatic pollutant-degrading bacteria, though without experimental confirmation [25]. Aromatic compounds are more commonly transported by proteins from other families which are secondary carrier-type facilitators. Most are in the major facilitator superfamily (MFS) including the Metabolite: H+ Symporter Family, the Aromatic Acid:H+ symporter (AAHS) Family, the Anion:Cation Symporter (ACS) Family, and the Putative Aromatic Compound/Drug Exporter (ACDE) Family. Additional families are the Aromatic Acid Exporter (ArAE) Family, the Hydroxy/Aromatic Amino Acid Permease (HAAAP) Family, and the Benzoate:H+ Symporter (BenE) Family.

#### Lipid-binding proteins

Many proteins exhibited stabilization by the addition of small molecule lipids as a primary function in the FTS assay. Seventeen periplasmic binding proteins shifted in the FTS assay with either medium/long-chain dicarboxylic acid’s (3 targets), medium/long-chain fatty acids (2 targets) or both (13 targets) (Table 2). There are at least three possible interpretations for these ligand-protein observations: 1) the lipid binding profile represents the functional role of the protein, 2) the binding is representative of an analog or cognate ligand, or 3) the association is the consequence of a nonspecific interaction. At this stage, it is not possible to define the nature of the interaction of most of the lipid-binding proteins in this study. However, some assignments can be corroborated by additional experimental studies and other assignments provide insight for refinement of binding studies or validation by alternative experimental approaches.

**Figure 3:**
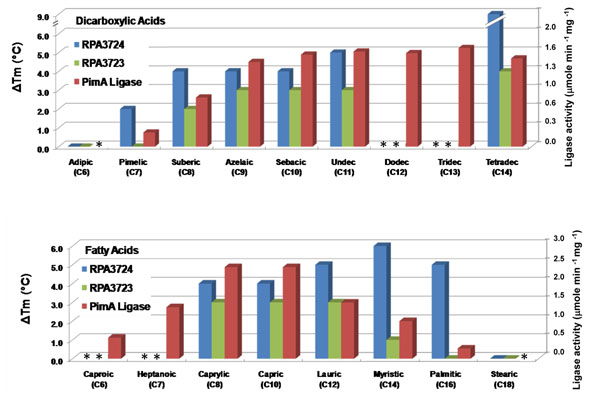
**Comparison of binding and enzyme activity profiles for selected proteins in or adjacent to the *pimFABCDE* operon. **The left ordinate represents increased stabilization increment (ΔT_m_) for the RPA3724 and RPA3723 binding proteins obtained in the presence of the indicated ligands relative to that obtained in the absence of ligands. Data represented is for dicarboxylic acid concentrations of 1000 µM assayed with 10 µM protein and fatty acid concentrations of 40 µM assayed with 4 µM protein.  Theright ordinate represents the activity of PimA ligase with dicarboxylic acid and fatty acid substrates as reported in the literature[26]. (*) Indicates protein was not tested with the respective ligand. Name abbreviations for fatty acids and C6-C10 dicarboxylic acids lack “acid” for brevity, while abbreviations for C11-C14 dicarboxylic acids are: undec , undecanedioic acid; dodec, dodecanedioic acid; tridec, tridecanedioic acid; tetradec, tetradecanedioic acid.

In particular, three SBP’s, RPA3725, RPA3724 and RPA3723, were examined more closely, since they are clustered in the genome with ABC transporter genes. RPA3725 was assayed but did not bind any ligand. RPA3724 and RPA3723 displayed different binding profiles for various fatty acids (FA’s) and dicarboxylic acids (DA’s). Binding profiles indicate that the two SBP’s together potentially function with the transporter to uptake a broad range of substrates having similar structures. This cluster of ABC transporter genes is adjacent to the *pimFABCDE* operon, the only identified operon to encode all enzymes for β-oxidation of fatty acids & dicarboxylic acids [26]. Enzymes in this metabolic pathway degrade medium chain DA’s (pimelate and longer) to glutaryl CoA and acetyl CoA. The *pimFABCDE* operon genes are required for optimum anaerobic growth on DA’s and are induced aerobically with C7-C14 DA’s and C8 FA. The pimA gene encodes acyl CoA ligase, which catalyzes the initial step of substrate activation with CoA. PimA ligase catalytic activity was characterized to have a broad substrate range for DA’s and FA’s. SBP profiles for DA’s correlate significantly to pimA ligase substrate preference (Fig. 3), which suggests that the likely function of this ABC transporter is broad range substrate uptake for degradation with these enzymes. This makes sense in the context of soil bacteria, since a significant source of environmental fatty acids and dicarboxylic acids is degradation products of suberin and cutin [27] which are major lipid constituents of plants.

The functional implication of remaining lipid-binding profiles is not immediately obvious. ABC transporters are involved in the transport and localization of lipids and their derivatives to the cytoplasm, periplasm and outer membrane as a consequence of anabolic and catabolic processes [28,29]. The transport of simple and complex phospholipids and glycolipids is mediated by ABC transporters, and these complexes are important for both the synthesis and recycling of cell wall components and membrane components [30,31]. However these lipid moieties are complex and for many outer membrane lipids the molecular form is species dependent. The current library contained only simple lipids and did not reflect the true complexity of the cellular lipid reservoir. In this context, it is reasonable to assume that some of the binding events observed in this system mimic components of the biological ligands. Delineation of the binding specificity will require expansion of the current screening library to increase representation of biologically relevant ligands and inclusion of additional experimental validation methods.

#### Amino acid binding proteins

Three proteins (RPA2499, RPA2628, and RPA3810) were identified as amino acid binding proteins via the FTS screening (Table 1). This observation is consistent with the assigned annotation and the general functional prediction of TransportDB (Table 2). The set of binding proteins specific for amino acids provide transport capabilities for seven of the individual amino acids. The genes encoding the RPA2628 (PBP), RPA2629 and RPA2630 (integral membrane subunits) (methionine/cysteine/histidine) are orthologs of a verified amino acid transporter in *Rhizobium leguminosarum*[*32*]. They are also localized in a cluster of 10 ABC transporter proteins containing four periplasmic binding proteins (three screened by FTS assay, Additional file 1) and RPA2628 was the only protein with specific ligand binding. The characterized set of binding proteins reflects an emphasis on maintenance of sulfate and nitrogen stores. RPA2499, annotated as an arginine binding protein, is adjacent to an “amidase” gene. Asparagine has a N:C ratio of 2:4, which makes it an efficient molecule for the storage and transport of nitrogen.

#### Phosphate/phosphonate binding proteins

Four proteins displayed stabilization with phosphate or phosphonates when screened using the FTS assay (Table 1). RPA0058 and RPA4780 exhibited specific stabilization by glycerol-3-phosphate and phosphate, respectively (Table 1). For both screened proteins, the assigned annotation and predicted substrate were in agreement and consistent with the experimental FTS assay. The RPA0699 and RPA0720 proteins had similar binding profiles and were stabilized by the addition of 2-aminoethylphosphonic acid, glycerol-3-phosphate, and phosphate. This observation is consistent with the assigned annotation for the RPA0699 protein and the general functional prediction of TransportDB (Table 2).

#### Urea and polyamine binding proteins

Urea and Polyamine content in cells is regulated by biosynthesis, degradation and transport. In *Escherichia coli*, there are two polyamine uptake systems, namely spermidine-preferential (PotABCD) and putrescine-specific (PotFGHI), which belong to the family of ATP binding cassette transporters. Similar to the *E. coli* protein, RPA2014, predicted to bind polyamines such as putrescine, spermine and spermidine, indeed shifted with putrescine preferentially over spermine (Table 1, spermidine was not assayed). The putative associated transporter subunits are elsewhere in the genome, annotated as *potH* (RPA4159) and *potI* (RPA4158) for the two integral membrane subunits and RPA4160 for the ATPase subunit. This SBP is an example of common occurrence in bacteria where “orphan” SBP’s are separated in the genome from the functionally associated transporter genes [33]. The SBP protein sequence is 51% identical to the *E. coli* putrescine binding protein (PotF) with solved structure in the PDB (1A99) and 38% identical to the *E. coli* spermidine binding protein (PotD) with solved structure in the PDB (1POT). Analysis of the respective ligands, putrescine and spermidine, in the binding sites for PotF and PotD reveal 7 key residues which confer specificity [34,35]. In PotF, these are Trp 37, Ser 38, Ser 85, Glu 185, Trp 244, Asp 247, Asp 278. All residues are identical or very similar in RPA2014 to PotF. PotF does not bind to spermidine or spermine due to two residues (Asp247 and Ser38) which affect N1 rigidity of the polyamine and are predicted to prevent the fit of longer compounds in the binding site. In PotD, one residue, corresponding to Asp247, is absent, resulting in more flexibility and space in the binding site for ligands longer than putrescine. Though these two residues (Asp247 and Ser38) are conserved in RPA2014 sequence, spermine still binds this protein with low affinity in the FTS assay. Based on these results, RPA2014 definitely plays a part in putrescine uptake, and may also transport spermine and spermidine, but further binding studies are needed to verify this observation.

### Comparison and analysis of predicted and experimental annotation for R. palustris SBPs

Despite fairly specific ligand predictions for most target proteins, only 11 of the 75 (14%) screened targets in the FTS assay exhibited binding consistent with the functional descriptions inferred from sequence homology or local genome context. These included proteins which bind urea (RPA1250, 3669), phosphate (RPA4780), sulfate (RPA0750), polyamine (RPA2014), nickel(II) (RPA2666), glycerol-3-phosphate (RPA0058), benzoate and related lignin monomers (RPA0668), di, tri, and oligo-peptides (RPA1218, RPA3691), metal cations (RPA0860, RPA0681), and phosphonic acid (RPA0699) (Table 2).

The remaining 37 targets with FTS assay ligand assignments displayed binding to a variety of compounds not predicted from sequence-based homology. Interestingly, very few of the 22 non-matching targets annotated as branched-chain amino acid or amide binding proteins (COG0683) exhibited a thermal shift with leucine, isoleucine, valine or short chain amides. Instead, these proteins bound combinations of other amino acids and/or various fairly hydrophobic compounds such as aromatic acids, medium to long-chain saturated fatty acids and/or dicarboxylic acids. Notably, 14 proteins bound ligands from more than one category and 9 of these were specific for dicarboxylic acids and fatty acids of similar structures. Only one protein, RPA3810, shifted primarily with amino acids with preferences for alanine, glycine and serine, and detectable but low affinity for leucine (Tables 2 and Additional file 3).

The other 15 targets were sorted into a variety of descriptive categories defined by COG numbers, as these designations are associated with fairly specific ligand classes for SBPs. A smaller set of 5 targets annotated as binding “nitrate, sulfate, thiamine, taurine and sulfonates” (COG0715) displayed specific affinity for compounds with multiple carboxylic acids and/or amino groups such as asparagine, malate, citrate, guanine, and thiamine. Uniquely represented in COG0687 (spermine/putrescine/polyamine) and COG3221 (phosphate/phosphonate) were RPA4648 and RPA1385 which bound p-coumaric acid and vanadate, respectively, instead of the predicted ligand classes. Also, single targets in each of the categories, COG0747 (di- or oligopeptides), COG0614 (Fe+3 siderophores, iron, cobalamine, hemin), COG0834 (lysine/arginine/ornithine), and COG1653 (sugar/maltose/trehalose, glycerol-3-phosphate), displayed affinity for long chain fatty acids, phosphates/phosphonates, metal cations, and peptides, respectively. Four proteins, assigned to either COG0845 or COG1463 as being associated with efflux of drugs, proteins, or organic toxic solvents exhibited moderate T_m_ shifts with aromatic compounds, zinc(II) and nickel(II).

There were 27 targets screened which did not bind any ligand in the FTS assay (Additional file 1). Ligand categories which were present in the assay’s library were indicated in the descriptions for 18 of these proteins including phosphate, iron, branched-chain amino acid, peptides, sulfonates, molybdate, and sugars or glycerol-3-phosphate. Since the ligand descriptions are fairly broad, it is likely that the specific representative library ligands in these categories are not the actual physiological ligands for these SBP’s, a situation remedied by expansion of the ligand library. Finally, nine targets had only general annotation as a “extracellular ligand-binding receptor”, “periplasmic solute binding” or “conserved hypothetical” protein, and are the most difficult to characterize without additional empirical data. Given the ~ 27 to 33 ligand categories present in the library, reflecting probable ligands from both predicted and experimental annotation, ~20 (66%) of these were represented in the positive functional assignments determined by the FTS assay. Predicted ligand categories likely to be identified but were not represented at all in screened target ligand profiles were nitrate and taurine/sulfonic acids.

## Discussion

There have been few experimental studies that characterize the transporter specificity on a genome scale and this study represents the first genome wide approach for the experimental characterization of ABC transporter proteins. The ligand screening approach identified binding ligands for 48 binding proteins associated with the set of ABC transporters. The overall ligand binding profile reflects the metabolically diversity of *R. palustris* and is consistent with characterized or inferred cellular metabolic capabilities as well as nutrient characteristics of the ecological niche. The FTS screen identified several binding proteins associated with transport of aromatic compounds and fatty and dicarboxylic acids. These capabilities are aligned with the characteristic of the isolation site, the subsurface layer of a forest litter pool, and the encoded genomic metabolic capabilities to enable utilization of structurally diverse compounds derived from degradation of plant material [21]. In many cases, these transporter complexes are co-located with clusters of genes associated with the biodegradation of aromatic compounds and fatty acids. The binding profiles for the aromatic transporters will provide a foundation for characterization of the substrate preference of the uncharacterized enzymes associated with aromatic compound degradation.

The FTS screen identified ligands and binding proteins associated with core cellular requirements of environmental organisms that reflect transport capabilities for metals, sulfate, phosphate, amino acids, peptide, and polyamines. For many of the solute binding proteins, the ligand binding assignments were supported by bioinformatic analyses or by the ability of the protein to bind chemically related ligands. Experimental observations that identified binding proteins for glycerol-3-phosphate, phosphate, sulfate, and peptides were consistent with sequence based predictions based on the original annotation or TransportDB. There was less overlap for experimental observations and sequence base predictions of metal, polyamine, vitamin, and amino acid binding proteins. The experimental screen confirmed some of the inferred binding properties but in other cases contradicted the assignment or provided a specific ligand assignment in place of a general prediction. This is not surprising in view of the limited number of binding proteins that have been characterized using biochemical or genetic methods. There are only a few categories of ligand binding proteins that have been experimentally characterized. Most of these studies examined a single or limited number of potential ligands and were not designed to examine the spectrum of the natural ligand diversity. This class of proteins also represents a challenge for specific functional annotation. Structural studies of this solute binding protein superfamily demonstrate these proteins generally exhibit a structure where two globular domains are connected by a hinge region that allows flexibility for “opened” and “closed” states [5]. The hinge region is variable in length and structure which influences the binding characteristics of the protein [36]. Particular residues in the binding sight are involved in ligand specificity, though rarely is there a large contiguous conserved sequence pattern that can be correlated to the binding site of an unknown protein without 3-D structural evidence or comparison to a homologous protein structure. Our results show many transporter solute binding proteins in *R. palustris* CGA009 are incorrectly annotated and suggest that functional characterization based on sequence analysis cannot be exclusively relied upon to predict biologically relevant and specific ligands. Given the best of predictions, there is still disparity between expected and experimentally determined function. As a case in point, the outcome for the three SBP’s, RPA3723, RPA3724 and RPA3725 varied greatly. While the former two proteins bound various fatty acids and dicarboxylic acids, RPA3725 did not bind any ligand. This is surprising, since these three genes are all very closely related and form a cluster that is specific to the *Rhodopseudomonas* and *Bradyrhizobium* clade. The protein sequence for RPA3725 is 71% identical to RPA3724 and 60% identical to RPA3723. The protein-ligand interactions identified in this study will enable improvement of sequence-based predictions and provide a basis for expanding our knowledge of transporter functionality.

The results for this genome set illustrate some of the limitations of the current system for functional characterization of these proteins and indicate areas to improve the scope of functional characterization. Approximately 74% of the genome set of solute binding proteins entered the functional screening component but candidate ligands were identified for only 64% of the screened proteins. These proteins were extensively dialyzed and exhibited thermal melting suggestive of a properly folded protein structure. Although we eliminate some attrition due to loss of function or protein-ligand co-purification, the disparity between the number of screened proteins and functional assignments is largely a reflection of the limited representation of the ligand library. In addition, some of the experimentally observed interactions represent noncognate ligand binding. The selection of chemicals for the screening library was not directed towards representation of binding proteins associated with efflux transporters [37], synthesis of cellular structures [31], or recycling of cellular biomolecules [38].

## Conclusions

This study provides experimental functional characterization for ABC transporter solute binding proteins from *R. palustris*. The ligand binding profiles and number of transport proteins specific for aromatic compounds is consistent with ecological and laboratory studies which demonstrate the capabilities of this organism for the utilization of plant degradation products such as lignin-derived aromatic compounds. The results of this study also provide important biological insight for the metabolic capabilities and environment fitness of this organism. This functional insight can be used to improve the annotation of related organisms and provides a route to evaluate the evolution of this important and diverse group of transporter proteins.

## Methods

### Target staging

Coding sequences for all ABC transporter associated solute-binding proteins (SBP’s) were extracted from the JGI Integrated Microbial Genomes page for *Rhodopseudomonas palustris* CGA009 and the protein sequences were characterized using the SignalP[39,40] and TMHMM [41] algorithms to identify hydrophobic sequence features and guide the selection of clonable regions. The peptides specified by cloned target sequences excluded predicted N-terminal signal peptide sequences and any overlapping N-terminal trans-membrane helices. Predicted N-terminal cleavage sites were manually adjusted for input into an automated primer design tool (http://tools.bio.anl.gov:8000/bioJAVA/jsp/PeriplasmicProteinDesign/index.html) that uses the predicted cleavage information derived from SignalP 3.0 to automatically generate amplification primers for the mature (cleaved) form of the protein.

### Gene cloning, protein expression and purification

The *E. coli* cloning and expression approach employed 96-well plate-based methods and parallel expression strategies [42]. Target genes were PCR amplified from genomic DNA (ATCC: *Rhodopseudomonas palustris* CGA009 # BAA-98D-5) using a KOD HiFi DNA polymerase reaction (Novagen) combined with a touchdown PCR program designed for the GeneAmp PCR System 9700 thermocycler machine (Applied Biosystems). The 50 µl reaction was enhanced for GC-rich genomic DNA by addition of 2.5% DMSO based on previous studies [43]. Amplification products included appended 5’ and 3’ ligation independent cloning (LIC) sites to enable simultaneous cloning in multiple vectors. To optimize protein solubility outcome for predicted periplasmic proteins, an initial set of 48 genes were cloned in parallel by a LIC method [44] into a two expression vectors, pMCSG7 and pBH31. While pMCSG7 expresses protein in the cytoplasm, pBH31 is used for periplasmic protein expression. Both vectors append an N-terminal hexahistidine (6x-His) fusion tag and TEV protease cleavage recognition sequence between the fusion tag and the target protein. Additionally, pBH31 codes for an N-terminal PelB leader sequence which is cleaved off during transport through the inner membrane into the periplasm [45]. Only 8 of 48 clones from the initial set were better expressed in pBH31; thus the remainder of the SBP target set was cloned only into pMCSG7 vector. Each target was characterized for amplification, expression, and solubility using 96-well plate assays and high-density gel formats for denaturing gel analysis of proteins [46]. Targets were scored as positive for expression and solubility if a detectable fusion protein of the correct molecular weight was observed on gels stained with Coomassie-based Simply Blue Safestain (Invitrogen). Targets scored as positive for solubility were sequence verified prior to purification and screening.

Clones expressing soluble proteins were scaled to 500 ml cultures and purified using standard affinity chromatography Ni-NTA bead (Qiagen) purification methods using a combination of the automated AKTA system (GE-Healthcare) as previously described [47] and parallel manual methods. All purified proteins retained the 6x-His tag, since previous investigation of the effect of the tag on the outcome of ligand binding detection revealed insignificant tag interference for this class of proteins [4]. The purified proteins were dialyzed for buffer exchange into an assay-compatible buffer, flash frozen in liquid nitrogen [48], and stored at -80 °C until the assay. Protein concentrations were initially determined by measuring absorbance at λ280 using UV-spectrophotometry then verified and assessed for purity by comparison with a BSA standard using SDS-PAGE denaturing gel electrophoresis and Simply Blue Safestain for protein visualization. Once thawed, proteins were stored at 4 °C and used within two weeks.

### Fluorescence-based thermal shift assay

#### Assay reaction components and set-up

An environmentally sensitive dye, SYPRO orange (Invitrogen, #S6650), was used at 5x concentration in all assays. Proteins were diluted to a standard concentration of either 5 or 10 µM, and screened with either 500 or 1000 µM ligand, respectively, to maintain an optimized 100x ratio of ligand to protein concentrations.

Absolute values for protein and ligand concentration did not significantly affect the outcomes of a particular reaction as long as the ligand to protein ratio was consistent. The conclusion was derived by analysis of a set of five positive control proteins with experimentally characterized values (ΔT_m_) for ligand stabilization. These targets were screened at both 5 µM protein with 500 µM ligand and 10 µM protein with 1000 µM ligand. The screening results indicated no significant difference (≤ 2 °C) in T_m_ shifts between reactions having different component concentrations but the same ligand to protein concentration ratio (data not shown). Test screens also indicated that this ratio is needed for optimal sensitivity for ligand binding detection. Exceptions to the standard 100x ratio were for reactions containing fatty acid ligands. These more hydrophobic compounds exhibited characteristic melt curves having fluorescence values higher than buffer background when screened with the dye at standard ligand concentrations. Reactions containing fatty acids were optimized to 40 µM ligand and 4 µM protein (10x ligand to protein concentration ratio) which decreased ligand background to minimal values, yet still enabled reasonably sensitive detection of ligand binding. Though high ligand background melt curve fluorescence was present for 500 µM or 1000 µM fatty acids, this did not always interfere with T_m_ shift interpretation in these reactions, especially for C8-C14 ligands. Thus, data for both 100x and 10x ligand-to-protein concentration ratios were collected and reported for targets which bound fatty acids (Additional file 3). Accordingly, T_m_ shift values displayed dependence on ligand concentration and are proportionally lower for 10x ratios. All protein and SYPRO orange dilutions resulted in final assay buffer concentrations of 100 mM HEPES, 150 mM NaCl, pH 7.5 in a standard 20 µl reaction volume. Reaction volume was 40 µl only if ligands used in the reaction were soluble as concentrated stocks in 100% DMSO and not aqueous buffer. Ligands in DMSO were added for a final reaction concentration of 2% DMSO.

#### Assay instrumentation, standard program parameters, data analysis

FTS assays were performed using two different quantitative PCR instruments. Initially, the first half of the target set produced was screened using an Mx4000 multiplex quantitative PCR instrument (Stratagene) that enabled thermal manipulations and dye fluorescence detection based on a previously published method [49]. Parameters employed were the same as those previously specified for program set-up and data analysis [4]. The remaining targets were screened using the more advanced LightCycler®480 Real Time PCR System (Roche). This enabled higher density, higher-throughput data collection by acquiring 30 points per degree over a 70 °C temperature range, decreasing assay time to 20 minutes, and allowing assay setup and program execution to be independent of plate configuration. Optimized run protocol parameters were defined in a “Protein Melt” program for 1 cycle using “Melting Curve” analysis mode (Table 3). Assay reactions were performed in 96-well, white PCR plates (Axygen, #PCR-96-FLT-W or Roche, #04729692001) and wells were capped using optical 8x-strip caps (Axygen, #PCR-2CP-RT-C) or optical sealing film (Roche, #04729692001). The instrument software monitors the fluorescence in real time, creating an output protein melting curve graph of temperature (°C) vs. fluorescence. Data was displayed and analyzed using the software’s “T_m_ calling” analysis option, which allows computation of the derivative curve displayed as temperature vs. –(d/dT) of the raw melt curve fluorescence values. From the derivative curve, the protein melting temperature midpoint (T_m_) was selected as the temperature corresponding to the minimum fluorescence value. The difference in shifts in melting temperatures (Δ T_m_, °C) between a protein with and without ligand indicated a change in the protein stability; positive Δ T_m_ for protein-ligand combinations was interpreted as potential ligand binding. Values for T_m_’s and Δ T_m_’s and were calculated objectively via an automated algorithm using Microsoft Excel software and raw data exported from the instruments.

To verify that using different instruments for the complete target set minimally affected the outcomes for ligand binding detection, a set of 10 positive control proteins with known ligands and Δ T_m_’s were screened with their respective ligands under identical reaction conditions in both quantitative PCR instruments (data not shown). Comparison of T_m_ values for protein alone indicated an average difference of 3 °C higher T_m_’s for reactions in the LightCycler®480 instrument versus the Mx4000 instrument. However, the difference between Δ T_m_ values generated on the two instruments for reactions containing protein and ligand was less than 1 °C. This indicates a systematic increase in all values of the protein melting profiles generated by the LightCycler®480 instrument, which does not significantly affect the computed Δ T_m_ values for comparable reactions with a specific target protein. Thus, the absolute Δ T_m_ values are independent of the two instruments utilized for this study.

#### Assay screening method

Proteins were screened using a two-step approach: an initial screen against all pools of ligands followed by a deconvolution analysis to determine individual ligand binding. Proteins displaying positive shifts of melting temperature midpoint (Δ T_m_) with certain pools in the initial screen were screened again with the pool and also with each individual ligand present in that pool to identify specific binding ligands. Most proteins were screened against all relevant pools to provide an equal opportunity for all proteins to bind all ligands. Proteins assigned to the various COG categories 0683, 0834, 0687, 0715, which were functionally characterized prior to expansion of the ligand library, are exceptions. All reactions in which pooled or individual ligands stabilized protein were independently duplicated, and averages of the duplicate Δ T_m_ values were reported. The maximum variability associated with each data point derived from averaged data of duplicate reactions was consistently less than 2 °C. In each plate experiment, negative control reactions were run for each protein with no ligand, for buffer only, and 5x SYPRO orange dye only. Fluorescence values for dye and buffer control reactions displayed no significant background thermal melting pattern compared to protein; thus, background was not subtracted from experimental fluorescence values since this correction did not affect T_m_ values. T_m_ values for all proteins were dependent on the buffer content, and for some proteins, the T_m_ value for a defined concentration differed significantly between reactions with and without 2% DMSO. In these cases, both T_m_ values were reported. All protein-only T_m_’s reported are an average of two of more replicates.

In the FTS assay, false negatives are expected if the native ligand is present but not detected. This can possibly result from loss of native protein structure, undetected ligand insolubility/instability, or the ligand–to-protein concentration ratio is too low to compensate for a low affinity binding or small protein/ligand complex stabilization. In the case of protein stability, 2 of 27 targets tested (RPA3961 and RPA0048) but with no ligand binding outcome did not display a clear thermal melt curve with fluorescent dye. These proteins may have been partially denatured prior to the assay, but were not repurified and retested. The remaining 25 targets were considered properly folded since a clear melt curve was reproducibly generated from fresh samples and the protein-only T_m_ value was consistent across replicates.

### Ligand stocks and ligand pools preparation

Individual ligands were dissolved in either buffer containing 100 mM HEPES and 150 mM NaCl, pH 7.5 (1x Standard HEPES) or 100% DMSO, depending on solubility, and stored at 4°C. Exceptions were guanine and hypoxanthine, which dissolved in 1x Standard HEPES buffer at pH 10, and diaminopimelate, which dissolved in 1x Standard HEPES buffer at pH 1.5. These ligands were added to the assay so that the final amount of buffer at nonstandard pH was 2%. The cysteine stock solution contained equimolar amounts of DTT to prevent oxidation during storage and assay. All ligands were purchased from Sigma-Aldrich-Fluka-Supelco, except Putrescine (MP Biomedicals), oleic acid (EMD Biosciences), histidine (Novabiochem), cysteine (J. T. Baker); anhydrous sodium thiosulfate, D(+)-maltose, D-xylose, and iron(III) chloride (Fisher Scientific); disodium molybdate dehydrate and cupric chloride dihydrate (Mallinckrodt); and tryptone digest, anhydrous glucose and anhydrous sodium phosphate (American Bioanalytical). Ligand pools included no more than 10 ligands each and were developed systematically based on ligand chemical classification and/or compatible solubility for ease of high-throughput screening (Additional file 2).

Ligands were considered to be stable if they were soluble in HEPES buffer or 100% DMSO at room temperature and pH 7.5. Many ligands demonstrated significant binding to more than one test protein indicating consistent, reproducible solution stability in the assay, or were previously assayed with positive control proteins (eg. amino acids). Ligands suspect of potential insolubility were those dissolved in 100% DMSO, which were added to the assay reaction as only 2% DMSO in HEPES buffer. No direct measurement was made to verify solubility in these cases except qualitative observation of precipitation or discoloration. All DMSO ligands were added to the reaction last and immediately before performing thermal denaturation to minimize insolubility. Ligand concentration in the standard reaction was 100-fold more than protein concentration to ensure reasonable detection of binding even for protein/ligand pairs with lower affinity.

## Authors' contributions

Conceived and designed the experiments: FC, SG, AF. Performed the experiments: SG, AF, CS, DC. Analyzed the data: FC, SG, AF. Contributed reagents/materials/analysis tools: FC LH . Wrote the paper: FC, SG, LH.

## Competing interests

The authors declare that they have no competing interests.

## Supplementary Material

Additional file 1 Summary of ABC Binding Protein Features and Screening Outcome. The file provides a list of the genome set of 105 candidate solute binding proteins from *R. palustris* that were examined in this study linked to the screening outcome for the FTA assay. The table includes the Gene Locus Tag, COG ID and the associated ATPase and/or membrane permease ABC transporter proteins. The transporter components are organize by their genome proximity to the solute binding protein and may not necessary be functionally related. Click here for file

Additional file 2 Composition of the Ligand Library Used for Screening with the FTS Method. Click here for file

Additional file 3 Ligand profiles for all solute binding proteins screened in the FTS assay. A list of all protein:ligand interaction observed using the FTS method. Click here for file
